# Colchicine in ischemic heart disease: the good, the bad and the ugly

**DOI:** 10.1007/s00392-021-01828-9

**Published:** 2021-03-13

**Authors:** Domenico D’Amario, Donato Cappetta, Luigi Cappannoli, Giuseppe Princi, Stefano Migliaro, Giovanni Diana, Karim Chouchane, Josip A. Borovac, Attilio Restivo, Alessandra Arcudi, Antonella De Angelis, Rocco Vergallo, Rocco A. Montone, Mattia Galli, Giovanna Liuzzo, Filippo Crea

**Affiliations:** 1 Dipartimento Di Scienze Cardiovascolari E Toraciche, Fondazione Policlinico A. Gemelli IRCCS, Università Cattolica del Sacro Cuore, Largo A.Gemelli 8, Rome, 00168 Italy; 2 Università Cattolica del Sacro Cuore, Rome, 00168 Italy; 3 Department of Experimental Medicine, University of Campania L. Vanvitelli, Naples, 80138 Italy; 4 Department of Pathophysiology, School of Medicine, University of Split, Split, 21000 Croatia; 5 Division of Cardiology, University of Florida College of Medicine, Jacksonville, FL USA

**Keywords:** Colchicine, Ischemic heart disease, Cardiovascular events, Tailored therapy, Efficacy and safety, Personalized medicine

## Abstract

Inflammation is the main pathophysiological process involved in atherosclerotic plaque formation, progression, instability, and healing during the evolution of coronary artery disease (CAD). The use of colchicine, a drug used for decades in non-ischemic cardiovascular (CV) diseases and/or systemic inflammatory conditions, stimulated new perspectives on its potential application in patients with CAD. Previous mechanistic and preclinical studies revealed anti-inflammatory and immunomodulatory effects of colchicine exerted through its principal mechanism of microtubule polymerization inhibition, however, other pleiotropic effects beneficial to the CV system were observed such as inhibition of platelet aggregation and suppression of endothelial proliferation. In randomized double-blinded clinical trials informing our clinical practice, low doses of colchicine were associated with the significant reduction of cardiovascular events in patients with stable CAD and chronic coronary syndrome (CCS) while in patients with a recent acute coronary syndrome (ACS), early initiation of colchicine treatment significantly reduced major adverse CV events (MACE). On the other hand, the safety profile of colchicine and its potential causal relationship to the observed increase in non-CV deaths warrants further investigation. For these reasons, postulates of precision medicine and patient-tailored approach with regards to benefits and harms of colchicine treatment should be employed at all times due to potential toxicity of colchicine as well as the currently unresolved signal of harm concerning non-CV mortality. The main goal of this review is to provide a balanced, critical, and comprehensive evaluation of currently available evidence with respect to colchicine use in the setting of CAD.

## Introduction

Inflammation is the main pathophysiological process involved in atherosclerotic plaque formation, progression, instability, and healing [1, 2]. For decades, a relentless search has been performed to find the most effective strategy to control and/or inhibit inflammatory pathways [3]. Ridker and colleagues, in the landmark CANTOS trial, proved for the first time in a clinical setting the robustness of the inflammatory hypothesis of atherosclerosis, showing that canakinumab, a fully human monoclonal antibody targeting proinflammatory interleukin-1β, causes a significant reduction in major adverse cardiovascular (CV) events (MACE) in patients with stable coronary artery disease (CAD) [4]. However, due to the observed increase in the number of deaths related to infections and pharmacoeconomic cost-effectiveness limited to high-risk patients only, widespread use of canakinumab did not show uptake in a contemporary clinical practice [5, 6].

When various antiinflammatory drugs were tested in the setting of CV disease, a relevant clinical impact in primary and secondary prevention was not achieved [7]. Methotrexate, as tested in the CIRT trial, failed to reduce MACE in patients with diabetes and stable CAD [8] while BI-204, a monoclonal antibody targeting a modified epitope of ApoB-100, did not achieve a reduction in biochemical and imaging markers of inflammation [9]. Darapladib, an inhibitor of Lp-PLA_2_, a phospholipase involved in the production of proinflammatory mediators was found to have no beneficial effect in patients with the acute coronary syndrome (ACS) and established CAD, as demonstrated in the SOLID-TIMI 52 [10] and STABILITY [11] clinical trials. Similarly, in the LATITUDE-TIMI trial, the p38-MAPK-inhibitor losmapimod failed to confer beneficial effects in patients with acute myocardial infarction (AMI) [12] while varespladib tested in ACS, despite its anti-inflammatory effects, actually increased the risk of AMI [13]. Encouraging results seemed to come from tocilizumab, a humanized anti-interleukin-6 receptor (IL-6R) antibody, tested in patients with AMI, but the clear impact on relevant hard end-points still seems far away to come [14, 15].

After the long streak of neutral and somewhat disappointing trial results with mostly novel pharmacotherapeutic agents, the idea of using colchicine, an old and well-known antiinflammatory drug, stimulated new research efforts and put it to a test in CV disease prevention [16].

## Colchicine: set the stage for a faithful old friend

Colchicine is a tricyclic lipophilic alkaloid derived from *Colchicum autumnale* (autumn crocus, meadow saffron) and *Gloriosa superba* (glory lily) where it is found in corn, seeds, and flowers. Known since antiquity, its use as a medicinal plant to treat joint pain was documented more than 3000-years ago in an old Egyptian medical papyrus, known as the Ebers Papyrus [17]. Later, Byzantine physician Alexander of Tralles recommended in his “Therapeutica” (A.D. 550) the use of *colchicum* (named hermodactyl or finger of Hermes) as a remedy for gout. Centuries of bad reputation followed, during which the drug was held in low esteem, as it was widely believed that its use was ineffective or even dangerous. There was a turning point in the late seventeenth and early eighteenth century when the use of colchicum in gout was reintroduced in Europe [18]. In 1819, Pelletier and Caventou isolated a substance from the roots of *Colchicum autumnale*, and in 1833, Geiger purified this substance and gave it the name of colchicine. In 1884, the pharmacist Alfred Houdé improved the purification process by obtaining pure crystallized colchicine compound [19].

### Chemistry

**Fig. 1:**
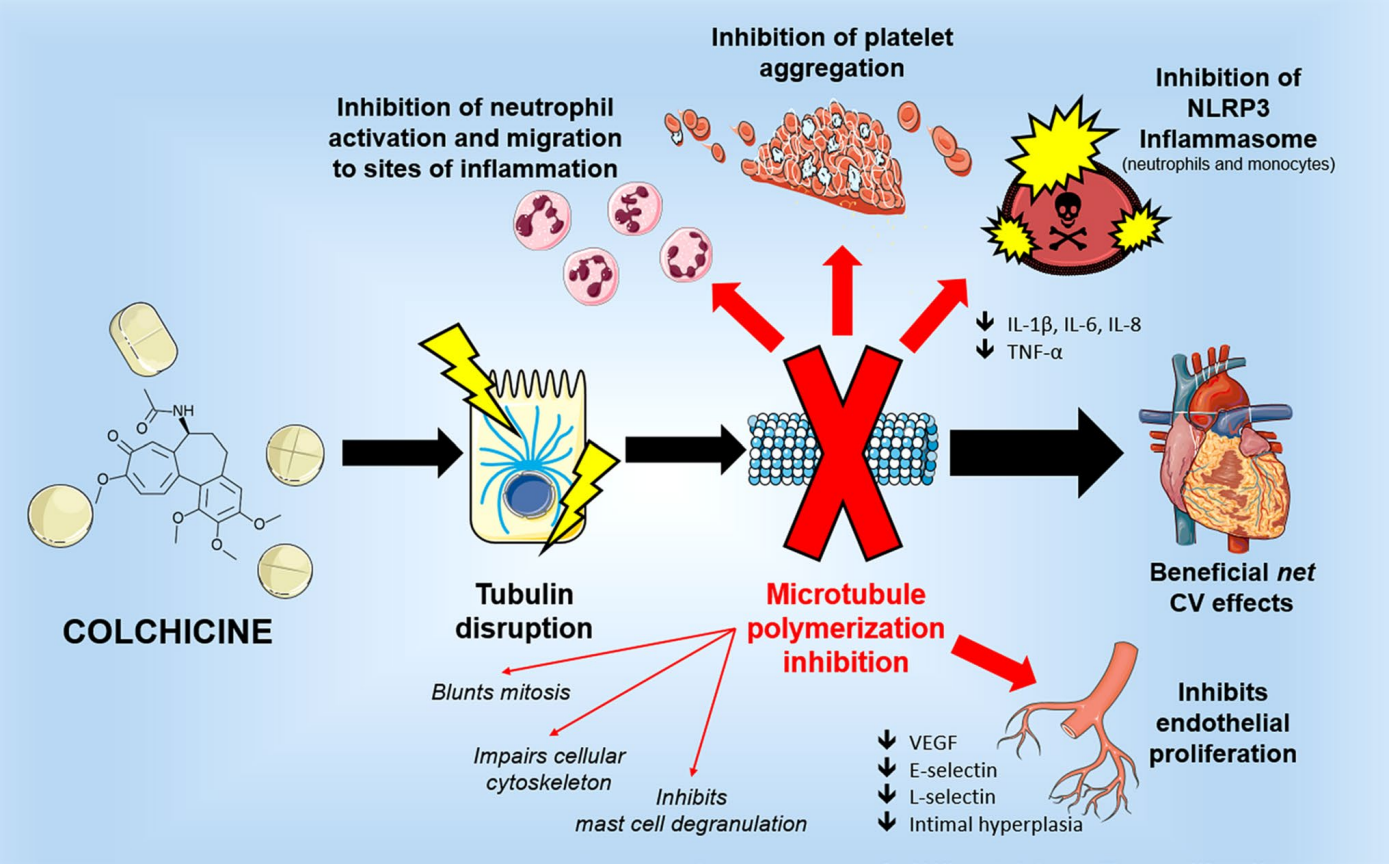
Colchicine mechanism of action—colchicine primarily causes tubulin disruption and prevents microtubule formation, thus resulting in neutrophils inhibition, antinflammatory effects, beneficial cardiovascular effects and inhibiting endothelial cells proliferation. *IL* interleukin; *NLRP3* NLR family pyrin domain containing 3; *TNFα *tumor necrosis factor alpha; *VEGF*  vascular endothelial growth factor

Colchicine is an alkaloid with the formula C_22_H_25_NO_6_; the chemical name is N-[(7S)-5,6,7,9-tetrahydro-1,2,3,10-tetramethoxy-9-oxobenzo(a)heptalen-7-yl)acetamide]. This molecule contains three rings: A-ring which is a trimethoxyphenyl moiety; seven-membered B-ring and C-ring which is a methoxytropone moiety (Fig. 1) [20]. A-ring and C-ring are held in a rigid configuration by B-rings and are highly involved in the binding to tubulin. Colchicoside, a colchicine analogue with a voluminous group in the A-ring, is no longer able to form a complex with tubulin. Similarly, the replacement of C-ring, in lumicolchicine and colchinol, and slight manipulation of the tropolone structure, in isocolchicine, produce inactive compounds with no affinity for tubulin [21, 22]. On the other hand, significant variations on the B-ring do not prevent colchicine from binding to tubulin but affect the interaction by modulating activation energy of the binding reaction and association/dissociation kinetics [23, 24].

### Pharmacokinetics

Colchicine is rapidly absorbed after oral administration and peak plasma concentrations occur within 1–2 h. It has a bioavailability that varies from 24 to 88% [25]. When released into the bloodstream, more than 40% is conjugated to plasmatic proteins and the formation of stable colchicine-protein complexes in many tissues contributes to its large volume distribution (2.2 L/kg). Colchicine accumulates in the inflammatory cells, with an intra-leukocyte concentration higher than plasmatic concentration. Although it can cross the placenta and distribute into the breast milk, no clinical impact on the infant’s health was observed, likely because of a very low concentration of colchicine in these compartments. Up to 20% of colchicine is excreted in the urine, while most of the drug undergoes enterohepatic recirculation and is excreted via bile and feces. The average elimination half-life is 20 h [26, 27]. Colchicine is a substrate for cytochrome P3A4 (*CYP3A4*) and P-glycoprotein (P-gp) efflux transporter that are largely responsible for its metabolism and elimination. Colchicine is subjected to extensive first-pass metabolism. Intestinal and hepatic CYP3A4 metabolizes colchicine through oxidative demethylation to 2- and 3-demethylcolchicine. P-glycoprotein extrudes colchicine from the gastrointestinal tract limiting gastrointestinal absorption [28] CYP3A4 and P-glycoprotein are largely responsible for colchicine’s drug-drug interactions. Severe adverse interactions have been reported in patients taking colchicine and P-gp inhibitors (*e.g.* cyclosporine, calcium channel blockers and ranolazine) or *CYP3A4* inhibitors (*e.g.* clarithromycin, fluoxetine, ketoconazole, nefazodone, and cimetidine), resulting in an impaired colchicine metabolism and a consequent elevation in colchicine plasma concentration and toxicity. If this occurs, a colchicine dose reduction is recommended [26]. Moreover, dose adjustment of colchicine is suggested in the elderly population and patients with renal or hepatic impairment [29].

### Pharmacodynamics

The mechanism of action in colchicine is not fully understood. Early studies identified the microtubule as the primary subcellular target. The interaction between colchicine and tubulin depends on temperature, pH and concentration of tubulin [29, 32]. There is a non-covalent, poorly reversible binding that occurs with a stoichiometry of 1:1 molar ratio of colchicine to tubulin dimer. According to the currently most used model, reversible binding is followed by slow conformational changes that converts the initial complex to a more stable, less easily reversible state [22]. Colchicine binds to tubulin heterodimers impairing spatial conformation, thus causing tubulin disruption and preventing any further microtubule growth [30, 31]. Dynamics of microtubule polymerization are essential to cellular functions, such as cell division and migration, intracellular organelle and vesicle transport, and the secretion of cytokines and chemokines [32]. The antimitotic effect against microtubule and spindle formation, although not the only one, is considered the major mechanism by which colchicine intervenes in the molecular processes underlying the inflammation of gout, which remains the most common clinical indication for the use of colchicine.

Colchicine induces down-regulation of multiple inflammatory pathways and the modulation of the innate immunity has most extensively been studied in the clinical context of gout, which is triggered by the deposition of monosodium urate crystals within joints [33]. This process causes an intensive inflammatory response characterized by a massive infiltration of neutrophils, macrophages/monocytes and dendritic cells, resulting in marked tissue injury [34, 35]. While colchicine does not interfere with urate crystal accumulation, it effectively modulates various actions of activated neutrophils that constitute a primary source of immune cells present in the synovial fluid and are pivotal in the generation of crystal-induced acute inflammation. Activated immune cells secrete multiple inflammatory cytokines, including tumor necrosis factor alpha (TNFα), interleukin (IL)-1β, IL-6, and IL-8 that, in turn, foster the increased expression of adhesion molecules on the surface of endothelial cells, such as E-selectin, and vascular cell adhesion molecule-1 (VCAM-1) [36]. In addition, urate crystals stimulate the production of superoxide anions from neutrophils necessary for the involvement of the nod-like receptor (NLR) family pyrin domain containing 3 (NLRP3) inflammasome complex that also has a recognized role in inflammatory gout attacks [37, 38]. As mentioned previously, the drug highly concentrates in immune cells where it impairs the secretion of cytokines thus attenuating the inflammatory response [26]. In addition, by modulating E-selectin expression on the surface of endothelial cells, colchicine further reduces neutrophil recruitment and by hampering vesicular trafficking, it is able to decrease both TNFα synthesis in macrophages and TNFα-receptor expression in both macrophages and endothelial cells [30, 39, 40]. Yet, disruption of the microtubular network could negatively affect several fundamental cellular processes. Besides the risk of multi-organ toxicity in the case of overdose, there are many known adverse reactions; particularly the most common are gastrointestinal (GI) symptoms including diarrhea, vomiting and nausea, while less common symptoms include myopathy, hematologic disturbances, and muscle weakness. Of note, colchicine may also have a direct toxic effect on cardiomyocytes interfering with its contractile and conduction properties. The main immunomodulatory effects of colchicine are shown in Fig. 1 and summarized in Table 1.

**Table 1:** Molecular and biological effects of colchicine and clinical implications

Colchicine mechanisms of action	Clinical implications
Disruption of tubulin and anti-mitotic effect (primary mechanism of action) Inhibition of the NALP3 inflammasome Inhibition of CASPASE-1 and downstream release of IL-1β Enhances dendritic cells maturation and their antigen presentation to naive CD4 + lymphocytes	Treatment and prevention of recurrent pericarditis and reduction of postpericardiotomy syndrome after cardiac surgery
	Prevention of atrial fibrillation incidence after cardiac surgery and recurrence after ablation
Inhibition of macrophages release of ROS, TNF-α, NO, and IL-1β Inhibition of neutrophil activation, mobilization, chemotaxis and release of IL-1β, IL-8, superoxide, chemotactic factors and L-selectin Inhibition of intimal hyperplasia and leukocyte VEGF expression in angioplasty model in dogs Inhibition of VEGF expression/release and endothelial proliferation Reduction of endothelial cells E-selectin expression and neutrophil adhesion inhibition (low doses)	Reduction of plaque inflammation, progression and rupture Prevention of acute cardiovascular events and restenosis after PCI is performed
Increase of Bcl-2 expression and suppression of Caspase-3	Reduces kidney tubulointerstitial fibrosis
Inhibition of TGF-β1 expression	Reduces peritoneal sclerosis

*Bcl-2  *B-cell lymphoma 2; *IL* interleukin; *NALP3*  NACHT-LRRPYD-containing protein 3; *NO* nitric oxide; *ROS*  reactive oxygen species; *TGF-β1* transforming growth factor beta-1; *TNFα *tumor necrosis factor alpha; *VEGF* vascular endothelial growth factor

With time, colchicine's therapeutic use was expanded, covering, in addition to gout, diverse conditions such as familial Mediterranean fever (FMF), systemic amyloidosis, pericarditis, atrial fibrillation (AF), scleroderma, Bechet’s syndrome, and Sweet’s syndrome. Although colchicine is a well-established antiinflammatory agent, mainly used to treat gout and FMF, it has historically demonstrated benefits in a variety of CV conditions including pericarditis, post-pericardiotomy syndrome, post-procedural AF, and chronic heart failure (HF) while, during the last decade, the investigational focus has shifted to examining the potential benefit of colchicine in CAD [41, 42].

## The good

In 2013 Nidorf and colleagues [43] conducted the *PROBE LoDoCo* trial to establish the effectiveness of continuous low-dose colchicine compared to placebo in decreasing the risk of cardiovascular events in patients with angiographically documented stable CAD, showing a reduced risk to develop ACS, cardiac arrest, and ischemic stroke as compared with a placebo. Seven years later Nidorf, with a new and more robust trial—*LoDoCo2* [44, 45] went on to further investigate the effect that 0.5 mg of colchicine administered daily had on patients with the chronic coronary syndrome (CCS), demonstrating a significant reduction in the composite outcome of cardiovascular death, spontaneous MI, ischemic stroke, or ischemia-driven coronary revascularization (31% lower relative risk, with a hazard ratio of 0.69, 95% CI 0.57–0.83).

In 2019 with their *COLCOT* trial Tardif and colleagues [46] assessed the effectiveness of colchicine in preventing major adverse cardiovascular events (MACE) in patients who experienced a recent MI. They showed that using 0.5 mg of colchicine daily significantly reduced the risk of cardiovascular death, MI, stroke, resuscitated cardiac arrest, or urgent hospitalization for unstable angina requiring revascularization during follow-up. Moreover, the effect of time-to-treatment on the primary and secondary efficacy outcomes showed that early initiation of low-dose colchicine within the first 3 days post-MI was associated with a reduction of 48% in the relative risk of MACE, compared with later initiation, such as on days 4 and 30. Furthermore, assessment of the in-trial period and lifetime pharmacoeconomic cost-effectiveness of low-dose colchicine treatment compared to placebo in post-MI patients on standard-of-care therapy [47] found that the mean overall *per*-patient costs were reduced by 47% for the in-trial and by 69% for the lifetime period and the incidence of diarrhea was comparable between groups (9.7% *vs* 8.9%* p* = 0.35).

Therefore, colchicine proved to reduce cardiovascular events in both acute and chronic CAD settings, *i.e.*, ACS in *COLCOT* and CCS in *LoDoCo2*. Although the pathogenic mechanisms underlying these beneficial effects have not been completely elucidated, it might be possible that early administration of colchicine in ACS acts by reducing ischemia–reperfusion damage [48], whereas its administration in patients with CCS promotes plaque stabilization and enhances plaque healing [49, 50]. Of note, a recent study suggests that low-dose colchicine favorably modifies coronary plaque microstructure, producing a more stable, fibrous plaque phenotype [51].

Other trials supported the use of colchicine in ACS and stable CAD, confirming the pathogenetic role of the inflammation [52–54] and the potential efficacy of colchicine in these clinical scenarios. Of importance, several studies demonstrated an increase of intracardiac production of the inflammasome-specific cytokines IL-1β, IL-18, and downstream IL-6 in patients presenting with ACS [55] and that acute colchicine administration was associated with a significant reduction in the transcoronary production of these cytokines [56, 57]. Furthermore, Tucker et al. found that the increased intracoronary levels of two chemokines—CCL2 and CX3CL1, observed in patients with ACS, are effectively suppressed by the colchicine administration [58]. The antiinflammatory properties in the ACS setting were also confirmed by Deftereos et al. [48], showing that in patients with STEMI, the administration of colchicine after diagnostic angiography with a loading dose of 2 mg (1.5 mg + 0.5 mg after one hour) followed by 0.5 mg twice daily for 5 days, markedly suppressed levels of biomarkers reflecting post–MI inflammatory response, in particular neutrophil count and C-reactive protein (CRP) that were associated with infarct size, which, in turn, is directly related to prognosis [59]. Furthermore, in the patient subgroup that underwent cardiac magnetic resonance (CMR) imaging in the study from days 6 to 9 after MI, treatment with colchicine was associated with smaller infarct size and reduced biomarker release.

Current ongoing trials are further investigating the role of colchicine in patients with ACS to confirm the beneficial properties of this drug (Table 2). Among those, *CLEAR SYNERGY (OASIS 9)* is comparing colchicine, spironolactone, and placebo in patients referred for percutaneous coronary intervention (PCI) after STEMI or selected high-risk NSTEMI with the primary aim to evaluate the incidence of cardiovascular death, recurrent myocardial infarction, or stroke [60]. *CLEAR SINERGY Neutrophil Substudy* [61] is assessing the effect of colchicine on neutrophil activation in STEMI patients. The investigators are examining clinical and genetic factors that determine heterogeneity in response to colchicine treatment. Another ongoing trial is *COVERT-MI*, which aims to investigate adverse left ventricular (LV) remodeling (primary endpoint, measured by CMR), infarct size, and microvascular obstruction reduction in patients with AMI undergoing PCI treated with colchicine at the time of revascularization and for the following 5 days [62]. The effectiveness of colchicine has also been investigated in patients with heart failure. Accordingly, Deftereos et al. [63] showed that the use of colchicine reduced circulating inflammatory biomarker levels (in particular high-sensitivity CRP and IL-6), and had favorable effects on left-ventricular remodeling, although this study failed to demonstrate significant improvement of functional status in patients with HF and reduced (< 40%) ejection fraction (EF), namely HFrEF population.

**Table 2:** Currently ongoing clinical trials studying colchicine in ischemic heart disease

TRIAL	NCT No	Study design	Clinical setting	Interventions	Outcomes
CLEAR SYNERGY (OASIS 9)	NCT03048825	Randomized, quadruple blinded, with factorial assignment	STEMI and high risk NSTEMI	Colchicine or spironolactone *vs.* placebo	Incidence of cardiovascular death, recurrent myocardial infarction, or stroke
CLEAR SINERGY Neutrophil Substudy	NCT03874338	Observational, prospective	STEMI	Colchicine	Soluble L-selectin; other soluble markers of neutrophil activity; markers of systemic inflammation
COVERT-MI	NCT03156816	Randomized, parallel, quadruple-blinded	MI	Colchicine *vs.* placebo	Infarct size (at CMR); LVEF; Microvascular obstruction
COPMAN	NCT04139655	Randomized, parallel, triple-blinded	MI and myocardial injury in non-cardiac surgery	Colchicine 0.6 mg/day *vs.* placebo	Incidence of Myocardial Injury after Non- Cardiac Surgery (MINS); Adverse Events; infectious complications
DRC-04	NCT03376698	Randomized, parallel, quadruple-blinded	T2DM and CAD	Colchicine 0.5 mg/day or Colchicine 0.25 mg/day *vs.* placebo	Change in serum hs-CRP, FMD, adhesive ability of white blood cell and plasma myeloperoxidase level

*CAD*  coronary artery disease; *CMR*  cardiac magnetic resonance; *FMD*  flow-mediated dilatation*; hs-CRP*  high sensitive C-reactive protein; *LVEF*  left ventricle ejection fraction; *MI*  myocardial infarction; *NSTEMI*  Non ST Elevation Myocardial Infarction; *STEMI* ST Elevation Myocardial Infarction

## The Bad

### Side effects of colchicine in cardiovascular trials and drug interactions

While the available evidence is globally skewed towards a positive effect of colchicine on ischemic cardiac outcomes, the trade-off between this effect and the risk of adverse reactions (even an increase in the cumulative number of non-cardiovascular deaths) requires further exploration and safety adjudication. Below we discuss adverse reactions reported in some of the major trials. For example, in the *COLCOT* trial [46], within the colchicine treatment group, 17.5% of patients had GI-related adverse effects while 0.9% experienced pneumonia (possibly related to immunosuppressive effects of colchicine). Similarly, GI-related adverse effects were also observed in nearly one-quarter (23%) of patients treated in the *COPS* trial [64], and as much as 7% of all patients had to discontinue the use of colchicine within 30 days since initiation due to this problem. In the *LoDoCo* trial [51], 11% of patients stopped treatment early due to intestinal intolerance and an additional 5% ended therapy late due to a range of possible side effects. In the *LoDoCo2* trial [45], myalgia occurred in 21% of patients in the colchicine group. In the small-sized *COLIN* trial [65], ten patients receiving colchicine reported digestive intolerance (43.4%) with clinical symptoms of diarrhea, nausea or vomiting and discontinuation of treatment was required for three patients (13.0%). Such safety data indicate that the side effects might be an important obstacle to the widespread and more extensive use of this drug in clinical practice. Moreover, it has been reported that combining colchicine with statin treatment might increase the risk of myalgia and, very rarely, acute rhabdomyolysis, especially among patients with renal impairment although literature reports cases of colchicine-induced rhabdomyolysis in patients without renal impairment [66]. In addition to side effects, another element that could hinder a widespread use of colchicine in the setting of CV disease are the numerous drug-to-drug interactions as summarized in Table 3.

**Table 3:** Major interactions of colchicine with common use CV and non-CV drugs

Drug (or class) interacting	Interaction	Collateral effects	Reference
Carvedilol	Increase colch*i*cine serum concentrations due to intestinal, renal and liver P-gp inhibition	Neuromyopathy, rhabdomyolysis, hepato- and nephrotoxicity, cardiotoxicity	[70]
Ranolazine			
Spironolactone			
Ticagrelor	Reduced colchicine clearance due to inhibition of CYP450 3A4, by which colchicine is metabolized	Colchicine toxicity (nausea, vomiting, diarrhea, fatigue, myalgia, paresthesia)	[74]
Digoxin	Increase concentrations of both drugs due to competitive inhibition of P-gp efflux transporter in the intestine, renal proximal tubule and liver	Rhabdomyolysis, digoxin and colchicine toxicity (arrhythmias, GI symptoms, fatigue, myalgia, paresthesia)	7[2]
Antiarrhythmic drugs			
Amiodarone	Increase colchicine serum concentrations due to intestinal, renal and liver P-gp inhibition	Neuromyopathy, rhabdomyolysis, hepato- and nephrotoxicity, cardiotoxicity	[70]
Quinidine			
Diltiazem	Coadministration with inhibitors of CYP450 3A4 may significantly increase the serum concentrations of colchicine, which is primarily metabolized by the isoenzyme	Myopathy, neuropathy, multiorgan failure, and pancytopenia	[71]
Verapamil			
Dronedarone			
Statins	Pharmacodynamic and pharmacokinetic interactions. HMG-CoA reductase inhibitors have in fact individually myotoxic effects (additive to those of colchicine) but are also substrates of the CYP450 3A4 isoenzyme and P-glycoprotein efflux transporter, thus competitive inhibition may occur resulting in increased drug absorption and decreased excretion	Muscle weakness and markedly elevated creatine kinase levels; myopathy up to rhabdomyolysis resulting in myoglobinuric and acute renal failure	[72]
Hydroxychloroquine	Additive pharmacodynamic risk of peripheral neuropathy	Peripheral neuropathy	[72]
Antibiotics			
Clarithromycin	Inhibition of the CYP450 3A4-mediated metabolism and P-glycoprotein (P-gp)-mediated colchicine transport by clarithromycin resulting in significantly serum colchicine increase	Myopathy, neuropathy, multiorgan failure, pancytopenia	[73]
Other Macrolides	Coadministration with inhibitors of CYP450 3A4 may significantly increase the serum concentrations of colchicine, which is primarily metabolized by the isoenzyme		
Ciprofloxacin			
Antiviral			
Darunavir/ Ritonavir			[73]
Boceprevir/Telaprevir			
Antimycotic			[71]
Fluconazole			
Ketoconazole			

*CV* cardiovascular; *GI*  gastrointestinal; *P-gp* glycoprotein P

## The ugly

All that glitters is not gold and, as previously elaborated, colchicine does have its ugly side. Unfortunately, not all of the evidence available have in fact fostered enthusiasm about the role of colchicine on cardiovascular events with some major studies yielding neutral or negative results. For instance, the *COLIN* trial [65] failed to show a reduction in inflammatory and myocardial injury markers as well as beneficial effects on adverse LV remodeling after one month of colchicine administration in patients with STEMI, even if there was a late administration after reperfusion and the loading dose was missed, suggesting that the treatment should be given as soon as possible to reduce reperfusion injuries associated with inflammation burden. A recent systematic review summarizing key trials of colchicine use in ACS concluded that colchicine is likely to reduce MACE in patients with ACS if it is administered for longer than 30 days while it seems to be ineffective if it is administered only pre-procedurally [67]. Furthermore, Tong and colleagues, with their very recent Australian-based *COPS* trial [64] failed to demonstrate an improvement in cardiovascular outcomes related to the use of colchicine in nearly 800 patients with ACS. The primary endpoint of all-cause mortality, readmission for ACS, ischemia-driven urgent revascularization and non-cardioembolic ischemic stroke was not reached, with no significant differences in these outcomes between the colchicine group compared to the placebo group.

The *COLCHICINE-PCI* trial [68], released in the first half of 2020, has provided rather disappointing results focusing on the role of this drug in the peri-interventional setting. The investigators showed that preprocedural administration of colchicine could have only a minor effect in reducing post-procedural inflammation, even though the trial yielded a formal negative result. This study randomized patients referred for possible PCI, who were given a colchicine loading dose of 1.2 mg plus 0.6 mg within 2 hours before PCI. The primary endpoint of PCI-related myocardial injury was the same between groups and no significant difference was observed in the composite outcome of death, MI and target revascularization at 30 days. Yet, the inflammatory biomarker substudy, proved that the increase in IL-6 and hs-CRP at 24 h post-PCI was significantly reduced in the colchicine group, while this difference between treatment groups was not significant one hour after the procedure. A summary of major published trials on colchicine and its cardiovascular implications is shown in Table 4.

**Table 4:** Main studies published on colchicine and its CV implications

TRIAL (year)	LoDoCo [43] (2013)	COLIN [65] (2017)	LoDoCo2[45] (2020)	COLCHICINE-PCI [68] (2020)	COPS [64] (2020)	COLCOT [46] (2020)
Patients enrolled	535 (473 male)	44 (35 male)	5522 (4676 male)	400 (374 male)	795 (632 male)	4745 (3836 male)
Median follow-up	3 years	1 month	28.6 months	1 month	12 month	22.6 months
Setting	CCS	ACS	CCS	ACS/CCS	ACS	ACS
Study design and aims	Randomized, prospective, observer-blinded endpoint trial to assess efficacy of continuous low-dose of colchicine treatment in patients with stable CAD in reducing CV events	Randomized, prospective, open-label, controlled trial to assess effect of colchicine plus OMT or OMT alone in STEMI patients	Randomized, controlled, double-blind trial to further assess the effect of colchicine in patients with chronic coronary disease	Randomized, double-blinded, placebo-controlled trial to determine the effects of acute preprocedural oral administration of 1.8 mg of colchicine on PCI-related myocardial injury	Randomized, double-blind, placebo-controlled trial to assess the effect of oral colchicine on CV events in patients presenting with ACS	Randomized, double-blind, placebo-controlled, investigator-initiated trial to assess the effects of colchicine on CV outcomes and its safety profile in patients with recent MI (within 30 days)
Colchicine dosing regimen	0.5 mg QD	1 mg QD for 1 month	0.5 mg QD	Acute preprocedural oral use of 1.8 mg of colchicine	0.5 mg BID for first month followed by 0.5 mg QD for 11 months	0.5 mg QD
Primary endpoint	Composite of ACS, fatal or nonfatal out-of-hospital cardiac arrest, or noncar- dioembolic ischemic stroke	CRP peak during the index hospitalization	Composite of cardiovascular death, spontaneous (non- procedural) MI, ischemic stroke, or ischemia-driven coronary revascularization	PCI-related myocardial injury	Composite of death from any cause, ACS (STEMI/NSTEMI/UA), ischemia-driven urgent revascularization and non-cardioembolic ischemic stroke	Composite of death from CV causes, resuscitated cardiac arrest, MI, stroke, or urgent hospitalization for angina leading to coronary revascularization in a time-to-event analysis
Secondary endpoints	Components of the primary outcome and the components of ACS unrelated to stent disease	Troponin peak, tolerance of colchicine, hospitalization duration, MACE at 1-month follow-up; cardiac remodeling	Composite of cardiovascular death, spontaneous MI, or ischemic stroke	MACEs at 30 days; composite of the earliest occurrence of death from any cause, nonfatal MI, or target vessel revascularization; PCI-related MI; change in plasma inflammatory markers concentration between baseline and post-PCI	Components of the primary endpoint and hospitalization for chest pain	Components of the primary efficacy end point; composite of death from CV causes, resuscitated cardiac arrest, MI, or stroke; total mortality in time to-event analyses
Primary endpoint reached	YES 5.3%—colchicine 16.0%—placebo HR 0.33 (95% CI 0.18–0.59) P < 0.0001	NO 29.03 mg/L – colchicine 21.86 mg/L – control group P = 0.36	YES 6.8%—colchicine 9.6%—placebo HR 0.69 (95% CI 0.57–0.83) P < 0.001	NO 57.3%—colchicine 64.2%—placebo P = 0.19	NO 24 events – colchicine (24/396) 38 events – placebo (38/399) P = 0.09	YES 5.5%—colchicine 7.1%—placebo HR 0.77 (95% CI 0.61–0.96) P = 0.02

*ACS*  acute coronary syndromes; *CAD*  coronary artery disease; *CI* confidence interval; *CRP*  C-reactive protein; *CV*  cardiovascular; *HR*  hazard ratio; *MI  *myocardial infarction; *NS* non-significant; *OMT* optimized medical therapy; *RR*  relative risk; *UA* unstable angina

Furthermore, worrying findings have emerged from some of the major trials regarding non-cardiovascular risks of colchicine. In the *COPS* trial [64], an alarmingly higher rate of total death was observed in the colchicine group as compared to placebo. Similar results emerged in the *LoDoCo2* trial [45], in which investigators state in the discussion of their manuscript that the observed non-significant incidence of non-CV death in the colchicine arm could have been due to chance, although an obtained hazard ratio of 1.51 is of potential concern. Nevertheless, a trend towards increased incidence of death from non-CV causes in the colchicine arm was present also in the two others main randomized clinical trials on the topic, *COLCOT* and *LoDoCo2* thus suggesting a possible direct cause-effect relation between colchicine and non-CV death rather than a relation due to the play of chance. Importantly, it should be noted that none of the published RCTs were formally powered to address the outcome of non-CV death. To overcome this limitation, a recently published focused meta-analysis [69] pooled data from the main trials on the topic, showing a significant increase of non-CV death among colchicine-treated patients as compared to controls at an average follow-up of 25.1 months (OR 1.55, 95% CI 1.10 to 2.17; *p* = 0.010). Moreover, a specific cause of death responsible for this excess of deaths has not been identified, underlining the need for further studies to shed light on the precise etiology and pathophysiological mechanisms supporting an unequivocal cause-effect relation between colchicine and non-CV deaths.

## Conclusions

Considering the growing body of evidence obtained from RCT data, the use of colchicine demonstrated an effective reduction in ischemic events in patients with acute and chronic coronary syndromes while providing a favorable cost/benefit ratio, particularly when administered on top of the current optimal medical therapy. Downsides to its use are narrow therapeutic index, potential long-term toxicity, and notable drug-to-drug interactions. Furthermore, its net clinical benefit still needs to be unequivocally proven due to the signal of harm with respect to non-CV death. Such challenges would ultimately need to be tested in studies that will be powered for long-term efficacy and safety endpoints. To fully envision the use of colchicine in the treatment of ischemic heart disease, a major effort should be performed by personalizing its use in terms of timing, duration of treatment, and dose, reevaluating over time the net clinical benefit of this strategy by taking into account the underlying severity of CV disease, patient comorbidities and use of concomitant medications. Therefore, a specific biomarker or a combination of tools able to predict the response to colchicine treatment is still missing but could add information not available from the clinical assessment and help in the decision process regarding the use of this drug in clinical practice [70]

From these considerations, it arises the question about colchicine whether it is better to be loved rather than feared, or feared rather than loved, paraphrasing what Niccolò Machiavelli wrote in the Prince [71]. It might perhaps be answered: use with caution in a selected population of patients in which a net clinical benefit has been consistently proven.

## Abbreviations

ACS: Acute coronary syndrome
AD: Anno Domini
AMI: Acute myocardial infarction
CAD: Coronary artery disease
CMR: Cardiac magnetic resonance
CRP: C-reactive protein
CV: Cardiovascular
FDG/-PET/CT: Fluorodeoxyglucose/-Positron emission tomography/computed tomography
FMF: Familial Mediterranean fever
HF: Heart failure
hs-CRP: High sensitivity C-reactive protein
IL: Interleukin
LV: Left ventricle
MACE: Major adverse cardiovascular events
MI: Myocardial infarction
MRI: Magnetic resonance imaging
NLRP3: NLR family pyrin domain containing 3
NSTEMI: Non-ST-elevation myocardial infarction
STEMI: ST-elevation myocardial infarction
TNFα: Tumor necrosis factor alpha
PCI: Percutaneous coronary intervention
